# MHC Class II and Non-MHC Class II Genes Differentially Influence Humoral Immunity to *Bacillus anthracis *Lethal Factor and Protective Antigen

**DOI:** 10.3390/toxins4121451

**Published:** 2012-12-05

**Authors:** Lori Garman, Eric K. Dumas, Sridevi Kurella, Jonathan J. Hunt, Sherry R. Crowe, Melissa L. Nguyen, Philip M. Cox, Judith A. James, A. Darise Farris

**Affiliations:** 1 Arthritis and Clinical Immunology Program, Oklahoma Medical Research Foundation, Oklahoma City, OK 73104, USA; E-Mails: lori-garman@omrf.org (L.G.); eric-dumas@omrf.org (E.K.D.); skurella@haemonetics.com (S.K.); sherry.crowe@pharmathene.com (S.R.C.); melissa-nguyen@ouhsc.edu (M.L.N.); pcox3@jhu.edu (P.M.C.); judith-james@omrf.org (J.A.J.); 2 Department of Microbiology and Immunology, University of Oklahoma Health Sciences Center, 940 Stanton L. Young Blvd, Oklahoma City, OK 73104, USA; E-Mail: jonathan-hunt@ouhsc.edu; 3 Departments of Medicine and Pathology, University of Oklahoma Health Sciences Center, 1000 Stanton L. Young Blvd, Oklahoma City, OK 73104, USA

**Keywords:** *Bacillus anthracis*, protective antigen, lethal factor, vaccine, antibody response, MHC class II, mouse, genetic background

## Abstract

Anthrax Lethal Toxin consists of Protective Antigen (PA) and Lethal Factor (LF), and current vaccination strategies focus on eliciting antibodies to PA. In human vaccination, the response to PA can vary greatly, and the response is often directed toward non-neutralizing epitopes. Variable vaccine responses have been shown to be due in part to genetic differences in individuals, with both MHC class II and other genes playing roles. Here, we investigated the relative contribution of MHC class II versus non-MHC class II genes in the humoral response to PA and LF immunization using three immunized strains of inbred mice: A/J (H-2k at the MHC class II locus), B6 (H-2b), and B6.H2k (H-2k). IgG antibody titers to LF were controlled primarily by the MHC class II locus, whereas IgG titers to PA were strongly influenced by the non-MHC class II genetic background. Conversely, the humoral fine specificity of reactivity to LF appeared to be controlled primarily through non-MHC class II genes, while the specificity of reactivity to PA was more dependent on MHC class II. Common epitopes, reactive in all strains, occurred in both LF and PA responses. These results demonstrate that MHC class II differentially influences humoral immune responses to LF and PA.

## 1. Introduction

Anthrax infection is caused by the Gram-positive, spore-forming bacterium *Bacillus anthracis*. The ease and severity of inhalational infection, resistance of infective spores to the environment, and release of spores by terrorist groups [1,2] make *B. anthracis* a potent bioterrorism threat. *B. anthracis *produces two virulence factors: a poly-γ-D-glutamic acid capsule and anthrax toxin [3]. Anthrax toxin is a tripartite AB toxin composed of a single B component, Protective Antigen (PA), and two A components, Edema Factor (EF) and Lethal Factor (LF). The combination of PA with LF or EF produces Lethal Toxin (LeTx) or Edema Toxin, respectively; these toxins are important for infection of the host [4] and can contribute to mortality even when antibiotics have killed all bacteria [3]. While PA is non-toxic alone, it is a potent immunogen, and protection from spore challenge correlates with the LeTx-neutralizing humoral response to PA immunization [5,6,7,8]. All current forms of the anthrax vaccine contain or express PA [9]. The United Kingdom-licensed vaccine, Anthrax Vaccine Precipitated, contains significant quantities of LF [7], and antibodies specific for LF have been shown to provide protection against toxin or bacterial challenge in several animal models [10,11,12,13,14,15,16]. 

The humoral immune response to anthrax vaccination varies considerably from individual to individual. Examination of the humoral immune response to PA in individuals who had received the United States-licensed vaccine, Anthrax Vaccine Adsorbed, revealed that over 40% of vaccinees fail to neutralize LeTx better than unvaccinated controls [17,18]. Moreover, 1 in 8 (12.1%, 62/512) individuals who possessed high titer (≥1:1000) IgG anti-PA antibodies failed to neutralize LeTx *in vitro *better than unvaccinated controls [18], indicating that some individuals may develop immunodominant non-protective immunity to anthrax vaccination. Mapping the fine specificity of the PA and LF response with immune sera has demonstrated that many antibodies bind to epitopes on PA or LF with high affinity, but are not necessarily protective against *in vitro* or *in vivo* toxin or spore challenge [13,17,18,19]; in fact, some monoclonal antibodies can enhance toxicity [20]. Because of this variation in PA or LF antibody response and neutralization capacity, we and others have explored potential genetic or environmental causes of poor response to the anthrax vaccine [18,21]. We observed that African American individuals are less likely to develop high titer PA antibodies as compared to matched European Americans. Three HLA DRB1-DQA1-DQB1 class II haplotypes have been associated with decreased antibody responses to PA in humans, including *1501-*0102-*0602, *0101-*0101-*0501 and *0102-*0101-*0501 [21]. These haplotypes accounted for most of the suggested association between HLA and anti-PA antibody titer in a recent genome-wide association study from the same group [22]. Other suggested associations with anti-PA antibody titer in that study occurred near the human genes *SPSB1* and *MEX3C* on chromosomes 1 and 18, respectively. The extent to which genetic polymorphisms, including HLA haplotype, might impact the fine specificity of the humoral response to anthrax vaccination is unknown.

Experimental animal models for anthrax infection and vaccination include mice, rats, guinea pigs, rabbits, and non-human primates. While rabbits and non-human primates are thought to recapitulate the human disease most closely, A/J mice are commonly used as an animal model of anthrax infection due to their enhanced susceptibility to the attenuated Sterne strain, a trait that is mediated by a natural deletion in the C5 component of the complement cascade [23]. In addition, the effects that LeTx has on macrophages and dendritic cells of A/J mice are similar to its effects on human cells [23]. Since both the production of antibodies from protein immunization and the fine specificity of those antibodies has been shown to be mouse strain dependent [24,25,26,27], we utilized available A/J and C57BL/6 mice congenic for the H-2 region to dissect the relative contribution of MHC class II and non-MHC class II genes to immunization with anthrax toxin components PA and LF. 

## 2. Results and Discussion

### 2.1. Magnitude of Serum LF IgG Response to Vaccination Is More Dependent on MHC Class II than Magnitude of Serum PA IgG Response

To evaluate the genetic effect of the MHC class II locus on vaccine responses to anthrax LeTx components, three inbred strains of mice were immunized with recombinant PA or LF proteins in a three-dose priming schedule. Strains A/J, B6.H2k, and B6 were used. Strain A/J (H-2a) is of *k* haplotype at all MHC II loci, including *Ab*, *Aa*, *Eb*, and *Ea*, while B6 (H-2b) is of *b* haplotype at the *Ab*, *Aa* and *Eb* loci and is null for *Ea*. Strain B6.H2k has the background genes of B6 but is congenic for the H-2 locus of AKR/J (H-2k)*,* having *k* alleles at all Class II loci and *b* alleles at the MHC Class *1b* locus. Thus, comparison of B6.H2k responses to A/J or B6 responses permits deduction of MHC class II versus non-MHC class II genetic effects on vaccination. Mice from all three strains were vaccinated with 100 µg of either recombinant (r)PA or rLF in complete Freund’s adjuvant on Day 0, then boosted at Days 10 and 24 with 50 µg at each immunization. Groups of control mice were vaccinated with PBS/adjuvant alone according to the same schedule. Blood samples for antibody testing and epitope mapping were collected from individual mice at Days 0 and 28. 

**Figure 1:**
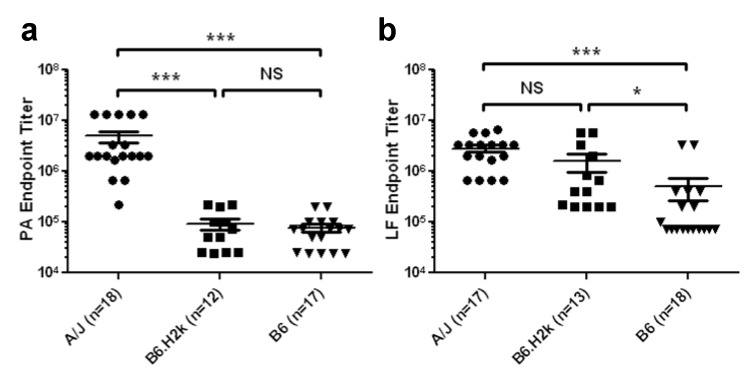
Magnitude of LF titer is more dependent on MHC II than that of PA titer after immunization. End-point serum IgG antibody titers to PA (**a)** or LF (**b**) in sera of groups of mice who had received three immunizations of rPA (**a**) or rLF (**b**) as determined by ELISA are shown. Positivity was defined as OD ≥ 3 SD above the average OD of pre-immunized serum diluted 1:100 from the same animals. Average OD (± SD) for pre-immunized mouse sera tested against PA was 0.068 ± 0.007 for A/J mice, 0.090 ± 0.023 for B6.H2k mice, and 0.120 ± 0.021 for B6 mice. Average OD (± SD) for pre-immunized mouse sera tested against LF was 0.102 ± 0.01 for A/J mice, 0.118 ± 0.01 for B6.H2k mice, and 0.119 ± 0.01 for B6 mice. Shown is the last titer at which each animal’s serum was positive, with the median for each group. *** *p* < 0.001; * *p* < 0.05 (Kruskal-Wallis test with Dunn’s post-hoc comparison). Data show combined results from two separate experiments.

First, to evaluate the influence of MHC class II genes on the magnitude of antibody responses to PA and LF, serum from each animal was diluted and tested separately for reactivity to the protein of immunization by standard ELISAs. All animals developed measurable antibody titers to the immunizing protein by Day 28. However, there was significant inter-strain variation in magnitude of the responses. PA-immunized A/J mice had significantly higher IgG titers to PA on Day 28 than B6.H2k or B6 mice (*p *< 0.001 for both comparisons), while responses of B6.H2k mice did not differ from that of B6 mice (Figure 1a). Averages and standard deviations of IgG anti-PA end-point titers of A/J, B6.H2k, and B6 mice were 4.95 × 10^6^ ± 5.26 × 10^6^, 9.36 × 10^4^ ± 7.78 × 10^4^, and 7.69 × 10^4^ ± 5.58 × 10^4^, respectively. LF-immunized A/J and B6.H2k mice responded with significantly higher anti-LF titers at Day 28 than B6 mice (*p* < 0.001 and *p* < 0.05, respectively; Figure 1b). Averages and standard deviations of IgG anti-LF end-point titers of A/J, B6.H2k, and B6 mice were 2.83 × 10^6^ ± 1.88 × 10^6^, 1.57 × 10^6^ ± 2.21 × 10^6^, and 5.01 × 10^5^ ± 1.02 × 10^6^, respectively. Interestingly, while titers to PA and LF in sera from A/J mice were similar, responsiveness to PA versus LF differed in both B6 (*p* = 0.013, Mann-Whitney *U* test) and B6.H2k mice (*p* = 0.0003, Mann-Whitney *U* test), suggesting that background genes outside of the MHC class II region can determine the magnitude of the response to a given immunogen.

### 2.2. Kinetics and Breadth of Response to PA Are Background Strain-Dependent

**Figure 2:**
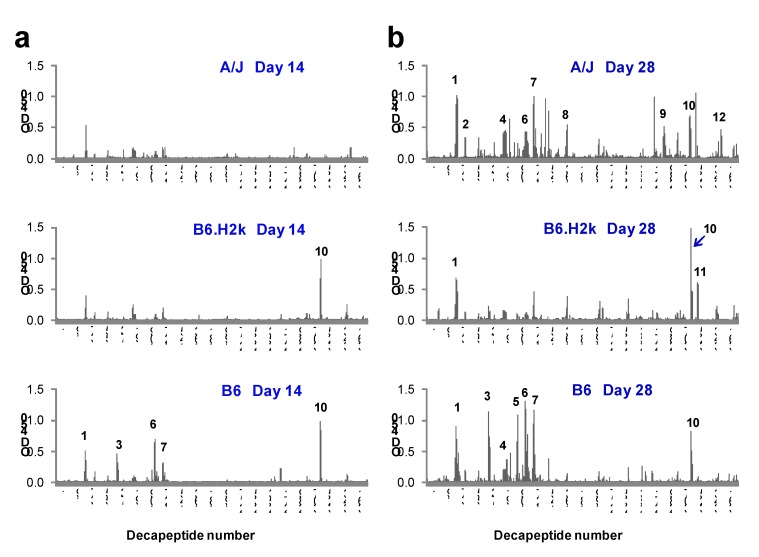
IgG anti-PA decapeptide binding increases with PA booster dose. Strain-specific serum samples from PA-immunized mice taken after the first (Day 14) or second (Day 28) booster immunizations were pooled and tested for binding to decapeptides of PA overlapping by 8 amino acids using solid-phase epitope mapping. Day 14 (**a**) and Day 28 (**b**) results are shown for A/J (upper panels), B6.H2k (middle panels) and B6 (lower panels) strains of mice. Epitopes are numbered in order from *N*-terminus to *C*-terminus using Day 28 data from all strains. Results shown are from one experiment. Similar results were observed in two independent experiments.

Strain- and immunization-specific Day 14 and 28 serum samples were then pooled and tested for sequential B cell epitopes by solid-phase epitope mapping. Day 28 serum samples pooled from PA-immunized mice recognized more PA decapeptides and epitopes compared to Day 14 samples from the same strains. At Day 28, A/J and B6 sera bound more epitopes than that of B6.H2k mice (Figure 2; Table 1). A/J mice reacted to a total of 9 epitopes, which occurred in all PA domains, and B6 mice reacted to 7 PA epitopes, which were located in domains I and IV. B6.H2k mice reacted to only 3 epitopes, and, like B6 mice, did not react to any decapeptides in Domains II or III. Some epitopes (1 and 10) were observed in all three strains and were thus designated as common.

**Table 1:** PA epitopes from three strains of inbred mice. Epitopes were defined by IgG binding to overlapping PA decapeptides in Day 28 serum samples and are numbered in order of occurrence from *N*-terminus to *C*-terminus of the PA amino acid sequence. Decapeptide number, amino acid sequence, amino acid number, and location within the PA domains are listed. Epitopes were classified as commonly occurring in all three strains (Common), determined by non-MHC class II genetic background (Background) or determined by MHC class II haplotype (MHC II) according to occurrence in particular strains as described in the text. Results shown are from one experiment. Epitopes 1, 2, 3, 5, 7, 8, 9, 10, 11 and 12 were confirmed in a separate, independent experiment.

Epitope Number	Decapeptide number	Sequence	Amino acid number	Domain	Reactive strain
1	36–38	DLSIPSSELENIPSENQY	71–84	I	A/J, B6, B6.H2k (Common)
2	47–48	IWSGFIKVKKSD	93–104	I	A/J (Background)
3	75–77	YQRENPTEKGLDFK	149–162	I	B6 (MHC II)
4	97–98	RKKRSTSAGPTV	193–204	I'	A/J, B6
5	110–111	EGYTVDVKNKRT	219–230	I'	B6 (MHC II)
6	120–122	IHEKKGLTKYKSSP	239–252	I'	A/J, B6
7	130–132	SDPYSDFEKVTGRI	259–272	I'	A/J, B6
8	170–171	HASFFDIGGSVS	339–350	II	A/J (Background)
9	287–289	GKDITEFDFNFDQQ	573–586	III	A/J (Background)
10	320–322	SVVKEAHREVINSS	639–652	IV	A/J, B6, B6.H2k (Common)
11	329–330	LLLNIDKDIRKIL	657–668	IV	B6.H2k
12	357–358	KLPLYISNPNYK	713–724	IV	A/J (Background)

Binding reactivity of Day 14 serum from PA-immunized A/J mice to PA decapeptides did not meet criteria for definition of any epitopes, though there was detectable reactivity to a single decapeptide within Day 28-defined Epitope 1. In contrast, the anti-PA response in B6 mice matured early, with 5 of 7 (71%) B6 PA epitopes bound at Day 14. Though B6.H2k mice reacted to only 3 epitopes at Day 28, positive reactivity to decapeptides within two of these (Epitopes 10 and a single decapeptide within Epitope 1) was observed at Day 14. Thus, B6 and B6.H2k strains mounted more rapid but ultimately less diverse responses to PA decapeptides than did the A/J strain.

### 2.3. Kinetics and Breadth of Response to LF Are Background Strain-Dependent

**Figure 3:**
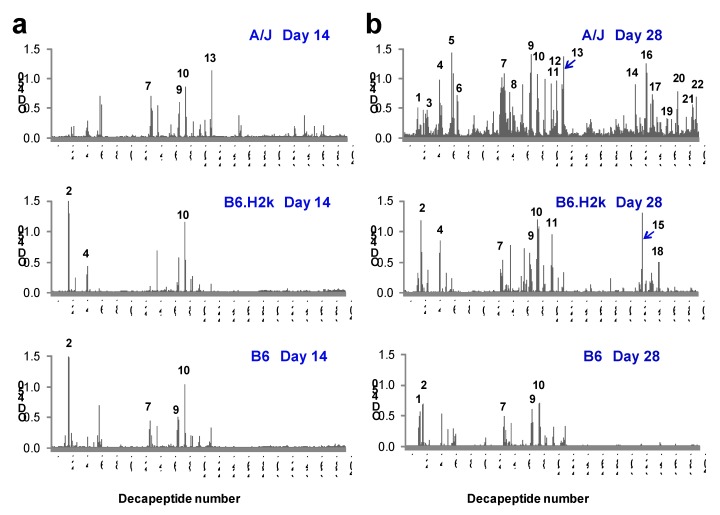
IgG anti-LF decapeptide binding increases with booster dose. Strain-specific serum samples from LF-immunized mice taken after the first or second booster immunizations were pooled and tested for binding to decapeptides of LF overlapping by 8 amino acids using solid-phase epitope mapping. Day 14 (**a**) and Day 28 (**b**) results are shown for A/J (upper panels), B6.H2k (middle panels) and B6 (lower panels) strains of mice. The cut-off for positive decapeptide binding was defined as the mean plus 10 SD of PBS/adjuvant-vaccinated control serum for all decapeptides. All other designations are as in Figure 2. Results shown are from one experiment. Similar results were observed in two independent experiments.

The fine specificity of serum IgG responses to LF in the three mouse strains was next evaluated. Pooled serum samples from LF-immunized A/J mice bound many more decapeptides compared to B6.H2k or B6 mice at both Day 14 and Day 28 (Figure 3; Table 2). By Day 28, A/J mice reacted to a total of 19 epitopes spread throughout all domains of LF. B6.H2k mice reacted to an intermediate number of epitopes (8) and, like A/J mice, targeted all four domains of LF. B6 mice reacted to the fewest LF epitopes (5) and displayed very little reactivity to epitopes in the *C*-terminus, which includes Domain IV and the second half of Domain II (Decapeptides 205–401).

IgG from Day 14 serum of all strains of mice bound fewer LF epitopes than their respective Day 28 samples. Specifically, Day 14 serum from A/J mice reacted to only 4 of the 19 LF epitopes recognized at Day 28 (21%; Epitopes 7, 9, 10, and 13; Table 2); however, there was some measurable binding that did not meet “epitope” criteria to decapeptides within an additional 2 epitopes eventually bound at Day 28. B6.H2k mice reacted to 3 of 8 (38%) B6.H2k Day 28 LF epitopes, and B6 mice reacted to 4 of 5 (80%) of B6.H2k Day 28 LF epitopes (Figure 3). Together, these results suggest that multiple vaccinations lead to greater diversity of the LF peptide-specific response in B6.H2k and A/J mice, while B6 mice react very similarly after a second LF booster as after a first booster. Interestingly, two of three commonly bound epitopes observed in Day 28 sera (Epitopes 9 and 10) demonstrated reactivity in all strains by Day 14, suggesting that they are common, immunodominant humoral epitopes. In addition, reactivity to the LF *C*-terminus observed in both A/J mice and B6.H2k mice at Day 28 was not present at all in either strain at Day 14, implying antibodies to this segment of LF generally develop late, if at all. 

**Table 2:** LF epitopes from three inbred strains of mice. Designations are as described for Table 1. Results shown are from one experiment. Epitopes 1, 2, 4, 5, 7, 8, 9, 10, 11, 13, 14, 15, 20 and 21 were confirmed in a separate, independent experiment.

Epitope number	Decapeptide number	Sequence	Amino acid number	Domain	Reactive strain
1	19–20	HGDVGMHVKEKE	37–48	I	A/J, B6
2	24–25	KEKNKDENKRKD	47–58	I	B6, B6.H2k (Background)
3	31–34	RNKTQEEHLKEIMKHI	61–76	I	A/J (Background)
4	49–51	EKVPSDVLEMYK	97–110	I	A/J, B6.H2k (MHCII)
5	66–70	SEDKKKIKDIYGKDALLH	131–148	I	A/J (Background)
6	74–75	LHEHYVYAKEGY	147–158	I	A/J (Background)
7	133–135	VLQLYAPEAFNYMD	265–278	I	A/J, B6, B6.H2k (Common)
8	149–151	MLARYEKWEKIKQH	297–310	II	A/J (Background)
9	172–174	SLSQEEKELLKRIQ	343–356	III	A/J, B6, B6.H2k (Common)
10	182–184	LSTEEKEFLKKLQI	363–376	III	A/J, B6, B6.H2k (Common)
11	201–203	LSEKEKEFLKKLKL	401–414	III	A/J, B6.H2k (MHCII)
12	209–210	QPYDINQRLQDT	417–428	III/II	A/J (Background)
13	216–218	LIDSPSINLDVRKQ	431–444	II	A/J (Background)
14	315–316	VESAYLILNEWK	629–640	IV	A/J (Background)
15	324–325	LIKKVTNYLVDG	647–658	IV	B6.H2k
16	328–331	LVDGNGRFVFTDITLP	655–670	IV	A/J (Background)
17	336–339	NIAEQYTHQDEIYEQV	671–686	IV	A/J (Background)
18	347–348	VPESRSILLHGP	693–704	IV	B6.H2k
19	358–359	EGFIHEFGHAVD	715–726	IV	A/J (Background)
20	372–373	NSKKFIDIFKEE	743–754	IV	A/J (Background)
21	391–394	DHAERLKVQKNAPKTF	781–796	IV	A/J (Background)
22	397–398	PKTFQFINDQIK	793–804	IV	A/J (Background)

In summary, this analysis demonstrates that the kinetics and diversity of the response to LF are strain-dependent, with the B6 strain making a rapid but less diverse IgG response.

### 2.4. Fine Specificity of the IgG Response to LF Is Strongly Influenced by Genetic Background

To remove any influence of differing magnitude of responses (Figure 1) from analysis of fine specificity, we re-analyzed the epitopes, assigning the 50 most reactive decapeptides of each strain (*i.e.*, those 50 with the highest OD values) as positive. Epitopes were again defined as two or more overlapping, positive decapeptides. Using this method, roughly similar numbers of epitopes were defined for each strain, effectively minimizing the influence of antibody titer on epitope selection (Table 3 and Table 4). By this method A/J mice have 12 PA epitopes and 13 LF epitopes, B6.H2k mice have 12 PA and 13 LF epitopes, and B6 mice have 10 PA epitopes and 10 LF epitopes. 

All defined PA epitopes were then classified according to the pattern of recognition by strain into those that are common among all strains regardless of genetic background (Common; Table 3), recognized only in A/J and B6.H2k mice or only in B6 mice and thus influenced by MHC class II haplotype (MHC II), or recognized only in A/J or in both B6 and B6.H2k strains and thus influenced by non-MHC class II genetic background (Background). Among the epitopes that could be classified into these groups 7 of 15 (47%) were commonly bound by all three strains, 5 of 15 (33%) were influenced by MHC class II genes, and 3 of 15 (20%) were influenced by non-MHC class II background genes (Table 3). For LF (Table 4), 9 of 17 epitopes were influenced by non-MHC class II background genes (53%), 4 of 17 were influenced by MHC class II alleles (23%), and 4 of 17 (23%) were common.

**Table 3:** PA epitopes defined by the 50 most reactive decapeptides. To eliminate bias from different levels of reactivity to the parent protein, epitopes were re-defined using the 50 most reactive decapeptides within each strain. Epitopes are numbered as in Table 1. New epitopes not defined in Table 1 are designated with letters. Classifications are as described in Table 1. Results shown are from one experiment. A separate, independent experiment confirmed that MHC class II-dependent PA epitopes are more frequent than non-MHC class II genetic background-dependent PA epitopes.

Epitope Number	Decapeptide number	Sequence	Amino acid number	Reactive strain
3	75–77	YQRENPTEKGLD	120–131	B6.H2k, B6 (Background)
9	287–290	GKDITEFDFNFDQQTS	544–559	A/J (Background)
12	357–358	KLPLYISNPNYK	684–695	A/J (Background)
				**3/15 (20%)**
1	35–38	TGDLSIPSSELENIPS	40–51	A/J, B6, B6.H2k (Common)
2	47–48	IWSGFIKVKKSD	64–75	A/J, B6, B6.H2k (Common)
4	93–97	LKQKSSNSRKKRSTSAGP	156–173	A/J, B6, B6.H2k (Common)
6	120–122	IHEKKGLTKYKSSP	210–223	A/J, B6, B6.H2k (Common)
7	130–131	SDPYSDFEKVTG	234–241	A/J, B6, B6.H2k (Common)
C	304–305	LDKIKLNAKMNI	578–589	A/J, B6, B6.H2k (Common)
10	320–322	SVVKEAHREVINSS	610–623	A/J, B6, B6.H2k (Common)
				**7/15 (47%)**
5	110–111	EGYTVDVKNKRT	190–201	B6 (MHCII)
8	170–171	HASFFDIGGSVS	310–321	A/J, B6.H2k (MHCII)
A	208–210	GKNQTLATIKAKEN	388–399	A/J, B6.H2k (MHCII)
B	273–274	TTKPDMTLKEAL	516–527	B6 (MHCII)
11	328–329	GLLLNIDKDIRK	626–637	A/J, B6.H2k (MHCII)
				**5/15 (33%)**
D	351–354	GKTFIDFKKYNDKLPL	672–687	B6.H2k

**Table 4:** LF epitopes defined by the 50 most reactive decapeptides. Designations are as in Table 3. Results shown are from one experiment. A separate, independent experiment confirmed that non-MHC class II genetic background-dependent LF epitopes are more frequent than MHC class II-dependent LF epitopes.

Epitope number	Decapeptide number	Sequence	Amino acid number	Reactive strain
1	19–20	HGDVGMHVKEKE	37–48	B6.H2k, B6 (Background)
2	23–25	KEKEKNKDENKRKD	45–58	B6.H2k, B6 (Background)
6	74–75	LHEHYVYAKEGY	147–158	A/J (Background)
C	145–146	LEELKDQRMLAR	289–300	A/J (Background)
E	191–193	SLSEEEKELLNRIQ	381–394	B6.H2k, B6 (Background)
11	201–203	LSEKEKEFLKKLKL	401–414	B6.H2k, B6 (Background)
12	209–210	QPYDINQRLQDT	417–428	A/J (Background)
20	372–373	NSKKFIDIFKEE	743–754	A/J (Background)
21	393–394	RLKVQKNAPKTF	785–796	A/J (Background)
				**9/17 (53%)**
4	49–51	EKVPSDVLEMYKAI	97–110	A/J, B6, B6.H2k (Common)
7	134–135	QLYAPEAFNYMD	267–278	A/J, B6, B6.H2k (Common)
9	172–174	SLSQEEKELLKRIQ	343–356	A/J, B6, B6.H2k (Common)
10	182–183	LSTEEKEFLKKL	363–374	A/J, B6, B6.H2k (Common)
				**4/17 (23%)**
A	109–110	DGQDLLFTNQLK	217–228	B6 (MHCII)
B	141–142	NEQEINLSLEEL	281–292	B6 (MHCII)
16	330–331	NGRFVFTDITLP	659–670	A/J, B6.H2k (MHCII)
17	336–339	NIAEQYTHQDEIYEQV	671–686	A/J, B6.H2k (MHCII)
				**4/17 (23%)**
3	32–33	KTQEEHLKEIMK	63–74	B6.H2k
5	66–69	SEDKKKIKDIYGKD	131–146	A/J, B6
D	167–168	IEPKKDDIIHSL	333–344	B6.H2k
13	216–217	LIDSPSINLDVR	431–442	A/J, B6
18	347–348	VPESRSILLHGP	693–704	B6.H2k

Thus, for humoral responses to PA and LF, both MHC class II-dependent and non-MHC class II genetic background-dependent B cell epitopes were identified. However, a dominating influence of non-MHC class II genetic background shaped the fine specificity of the humoral immune response to LF, while sequential epitopes of PA were more heavily influenced by the MHC class II locus. That is, the majority of all classified LF epitopes associated with non-MHC class II genetic background, *i.e.*, these epitopes were observed only in A/J mice or in only B6 and B6.H2k mice. 

### 2.5. Discussion

One of the undisputed goals of vaccination is to induce protective immunity in as many immunized individuals as possible. We have observed a wide variation in the magnitude and fine specificity of immunity to PA in humans who have received the AVA vaccine according to the originally recommended schedule [17]. Understanding genetic, environmental and stochastic contributors to this variation is key for developing strategies to induce protective immunity in a higher proportion of vaccinees. A portion of vaccine responsiveness is expected to be genetically determined, and one of the most important genetic contributors to vaccination is MHC class II haplotype. Indeed, three different human HLA class II DRB1-DQA1-DQB1 haplotypes were recently observed to associate with a reduced magnitude of antibody responsiveness to PA following AVA vaccination [21,22]. Other genetic effects of vaccine responsiveness are likely to be determined by non-MHC class II genes. Although the magnitude of the IgG antibody response to PA correlates with protective immunity as estimated by *in vitro* LeTx neutralization [5,6,8,14,17], we have documented a substantial number of AVA vaccinees who produce high levels of anti-PA IgG antibodies yet fail to effectively neutralize Lethal Toxin *in vitro* [17,18]. In addition, many antibodies directed toward PA or LF that are generated from mouse and human immunization are non-neutralizing [28,29], and certain antibodies to PA can actually enhance the effect of LeTx [20]. These observations highlight the importance of both fine specificity and magnitude of antibody responses in contributing to protective responses following vaccination. 

This study used inbred and MHC class II congenic mice to investigate the relative influence of MHC class II and non-MHC class II genes on both the magnitude and fine specificity of the humoral immune response to PA and LF. Our laboratory has previously reported sequential B cell epitopes of LF [13], EF [30] and PA [31] in A/J mice, but this is our first report of PA epitopes in B6 or B6.H2k mice. The previous reports demonstrated that identification of sequential B cell epitopes in mice is highly reproducible, in terms of both epitopes identified and relative magnitude of responses to individual epitopes, if pools of serum from groups of immunized mice are evaluated. All strains of mice in this study made robust antibody responses to both immunogens as detected by ELISA; however, strain-based variation in both the magnitude and fine specificity of the responses was observed. In particular, the MHC class II locus influenced the IgG titers to LF to a much greater extent than IgG titers to PA. Moreover, in the case of the LF response, the effects of MHC class II on magnitude and fine specificity were dissociable. In particular, MHC class II genes had a profound effect on LF antibody titer, whereas non-MHC class II background genes influentially shaped LF epitope selection. 

Considering the number of protein domains recognized, B6 mice made the most restricted response to both immunogens. While A/J mice responded to epitopes within all domains of PA and LF, B6 and B6.H2k mice had no reactive epitopes within PA Domains II or III. B6 mice also had no epitopes within LF Domains II or IV, while B6.H2k mice responded to two epitopes within Domain IV of LF. Thus, the overall breadth of response to PA was controlled primarily by background genes, and at least some of the breadth of response to LF was controlled by the MHC class II locus. The more restricted responses of B6 mice may be secondary to the lack of a functional I-E heterodimer owing the lack of an *Ea* gene in this strain. This situation might be relevant to humans who are homozygous for certain HLA class II haplotypes, a form of restriction of HLA diversity. In support of this idea, prolonged reductions in PA antibody titer were observed only in AVA vaccinees homozygous for HLA alleles associating with low-response phenotypes [21].

An analysis of strain-specific PA antibody response has been done in a similar manner once before without the use of H-2 congenic mice [32]. In contrast to the present study, vaccinations in that study were given intraperitoneally. The initial vaccination was given in CFA and the booster vaccinations were given in IFA. In addition, mice were only given 1 μg of PA at each dose, and PA-specific antibody titer was measured after initial immunization and 14 days after a single booster of PA. These authors found that B6 mice produced much higher PA antibody titers than A/J mice at this time point. While we measured antibody titers by ELISA only at the Day 28 time point, after two boosters, our Day 14 epitope mapping data, collected after only a single booster (Figure 2), is consistent with the results of this previous study. In particular, serum from B6 mice bound multiple epitopes at Day 14, while significant decapeptide binding by IgG in A/J mouse serum did not appear until Day 28, following two boosters. The kinetics of PA decapeptide binding in serum from immunized B6.H2k mice was intermediate between these two strains. LF decapeptide binding by serum from B6 mice also appeared to mature earlier than that of the other two strains, though the final responses of both B6 and B6.H2k strains were more restricted than that of A/J mice. These data suggest that genetic background can determine the kinetics of a vaccine response and are consistent with the need for a full priming series for timely, effective vaccination of a given population against anthrax. 

Notably, known neutralizing PA epitopes were only recognized by select strains of mice. That is, the furin cleavage site of PA amino acids (aa) [33,34,35] between Domain I’ and I [17] (here, PA Epitope 4) was not recognized as an epitope by B6.H2k mice, although some minor reactivity was detectable. The Domain II neutralizing PA epitope of the 2β2–2β3 loop [36] was targeted by A/J mice (here, Epitope 8) but was not bound by serum from B6 or B6.H2k mice, indicating that this is a background-sensitive epitope. We have previously shown that human antibodies affinity-purified against two PA epitopes (here, Epitopes 7 and 11) are protective against toxin challenge following passive transfer to A/J mice [17]. Herein, Epitope 7 is bound by A/J and B6 mice, while Epitope 11 is bound only by B6.H2k mice. Similarly, neutralizing LF epitopes were not always recognized by all three strains. We have previously demonstrated that human antibodies directed toward a peptide immediately preceding LF Epitope 8 are protective against toxin challenge following passive transfer into A/J mice [18]; in the present study, this epitope is only recognized by A/J mice. Neutralizing LF monoclonal antibody LF8 binds to decapeptides of LF [13] that correspond to Epitopes 9 and 10, which are recognized by all three strains. Another neutralizing LF monoclonal antibody, 9A11, binds a linear epitope that overlaps with Epitope 15, only recognized by B6.H2k mice, as well Epitope 22, bound by A/J mice. 

Evaluation of genetic influence on epitope specificity in this work has focused on sequential B cell epitopes. While the studies discussed above have shown that antibodies directed to such epitopes contribute to LeTx neutralization and/or protection following anthrax vaccination, they may form only a minor portion of the overall humoral response. Another consideration is whether choice of adjuvant has any impact on genetic control of humoral immunity to PA and LF. Nevertheless, these data demonstrate that genetic background exerts an important influence on humoral immune responses to PA and LF in a manner that may impact protection. While several studies have advocated vaccinating with specific peptides of PA [36,37], these data underscore the need for selecting multiple epitopes that are commonly targeted by individuals of diverse genetic background and HLA haplotype. 

## 3. Experimental Section

### 3.1. Animal Use

Inbred mice (6–8 week old) were obtained from the Jackson Laboratories (Bar Harbor, ME, USA) and maintained in the Oklahoma Medical Research Foundation (OMRF) vivarium. Strains included A/J (Stock #000646), B6.AK-H2k/J (B6.H2k, Stock #001895), and C57BL/6J (B6, Stock #000664). Animal protocols were approved by the OMRF Institutional Animal Care and Use Committee.

### 3.2. Production of Recombinant(r)PA and rLF Proteins

rPA and rLF were produced as amino-terminal 6× His-tagged proteins [13,38]. Briefly, BL21 *Escherichia coli* were transformed with pET15b expression vectors coding for full-length PA or LF. Protein expression was induced with 1 mM isopropyl β-D-thiogalactoside. Cells were disrupted with a French Press. Proteins were affinity purified from supernatants with Ni^2+^-charged affinity resins. Concentrations and purities of eluted proteins were determined by standard Bradford assays (Bio-Rad, Hercules, CA, USA) and electrophoresis in SDS-polyacrylamide gels followed by Coomassie blue staining.

### 3.3. Immunization and Blood Sampling

Mice from all three strains (Experiment 1: *n* = 10 female mice/group for A/J and B6, *n* = 5 female mice/group for B6.H2k; Independent Experiment 2: *n *= 8 male and female mice/group for A/J, B6 and B6.H2k) were vaccinated subcutaneously with 100 µg rPA or rLF mixed 1:1 in complete Freund’s adjuvant (CFA; Difco, Lawrence, KS, USA) on Day 0, then boosted subcutaneously on Days 10 and 24 with 50 µg rPA or rLF mixed 1:1 in incomplete Freund’s adjuvant (IFA; Difco) each time. Control groups of A/J and B6 mice (*n* = 10 mice/group for Experiment 1; *n* = 8 mice/group for Experiment 2) were vaccinated with PBS/adjuvant alone according to the same schedule and route. Blood samples for antibody testing and epitope mapping were collected from individual mice on Day 0 and 4 days after each boost by either retroorbital or cheek pouch sampling. Blood was allowed to sit at room temperature for 2 h, then centrifuged at 10,000 rpm for 10 min for serum isolation.

### 3.4. Recombinant Protein ELISAs

Ninety-six well plates were coated overnight at 4 °C with 1 μg/well of rLF or rPA (List Biologicals, Campbell, CA). After washing with PBS-Tween and blocking with PBS/BSA, diluted pre-immune and Day 28 (4 days after a second booster) serum from each mouse was added in duplicate and incubated for 2 h at room temperature (RT). After washing, the plates were incubated with Alkaline Phosphatase-labeled anti-mouse IgG (Experiment 1: 1:10,000 dilution, Jackson ImmunoResearch Laboratories, West Grove, PA, USA; Experiment 2: 1:5000 dilution, Sigma Immunoresearch) for 2 h at RT, washed again and incubated with pNPP substrate (Sigma, St. Louis, MO, USA) for 30 min. The optical densities (OD) at 410 nm were measured using a Dynex MRX II microplate reader (Dynex Technologies, Chantilly, VA, USA) and normalized to antigen-specific monoclonal antibodies present on each test plate. Positivity was defined as an average OD of greater than 3 standard deviations above the mean OD of serum samples from all pre-immune mice diluted 1:100.

### 3.5. Solid Phase Epitope Mapping

Pools of sera were created for each mouse strain, both for Day 14 and Day 28 and screened at a dilution of 1:200 for sequential B cell epitopes by solid-phase epitope mapping. Peptides, 10 amino acids in length and overlapping by 8 amino acids, covering the entire length of the LF or PA protein (Pub Med Protein accession numbers AAY15237.1 and AAA22637) were covalently synthesized onto polyethylene solid phase supports in a 96-well format as previously described [39,40,41]. The analysis for each strain of mouse was carried out separately, and then the epitopes were compared. For longitudinal analysis within-strain, epitopes were defined as two or more overlapping decapeptides having an OD of the mean plus ten times the standard deviation of PBS/adjuvant-vaccinated control serum tested against all decapeptides (positivity cut-off OD = 0.299). Due to significant disparities in antibody titers between strains, epitopes were re-defined to permit inter-strain comparisons in a manner that minimizes the influence of variation of antibody titer. Specifically, epitopes were re-defined as two or more overlapping decapeptides within the highest 50 peptide binding values recorded for that strain.

### 3.6. Statistical Analysis

ELISA titers between strains were compared by Kruskal-Wallis test, with reported *p*-values from Dunn’s post-hoc test. PA and LF ELISA titers within-strain were compared by the Mann-Whitney *U* test. Threshold positivity values for decapeptide binding were determined as described above. 

## 4. Conclusions

Using experimental mice, this study demonstrates that MHC class II haplotype and non-MHC class II genetic background can have divergent effects on both the magnitude and fine specificity of antibody responses, depending upon the immunogen studied. Despite these genetic influences, however, these results demonstrate the occurrence of protective epitopes commonly recognized by individuals of diverse HLA haplotype. We propose that such epitopes should be selectively incorporated into peptide-based vaccination strategies. This study further highlights the importance of conducting genetic association studies of vaccine responses using readouts that take into account not only general antibody levels directed to the immunogen of interest but also fine specificity of protective antibody responses. This might be best accomplished by utilizing toxin-neutralizing antibody titer as a critical phenotypic trait. 
